# Assessing Cardiometabolic Disease Risk Factors Among Healthcare Workers in a Rural Tertiary Care Hospital in Wardha, India: A Study Protocol

**DOI:** 10.7759/cureus.63261

**Published:** 2024-06-27

**Authors:** Mayank Sharma, Abhay Gaidhane, Sonali G Choudhari

**Affiliations:** 1 Community Medicine, Jawaharlal Nehru Medical College, Datta Meghe Institute of Higher Education & Research, Wardha, IND

**Keywords:** india, cross-sectional study, risk factors, rural setting, healthcare workers, cardio-metabolic diseases

## Abstract

Background

Cardiometabolic diseases pose a significant public health challenge globally, particularly among healthcare workers, who often face heightened occupational stress and lifestyle challenges. This study aims to assess the prevalence of cardiometabolic risk factors and their determinants among healthcare workers at Acharya Vinoba Bhave Rural Hospital, a tertiary care hospital in rural Wardha, Maharashtra, India.

Methods

A cross-sectional study design was employed, involving the recruitment of healthcare workers from various job roles. Data on demographic characteristics, behavioral risk factors, anthropometric measurements, biochemical parameters, and mental health status was collected using standardized instruments and procedures. Statistical analysis included descriptive statistics, inferential tests, and multivariate analyses to identify significant associations and predictors of cardiometabolic risk factors.

Expected results

Anticipated findings include a notable prevalence of cardiometabolic risk factors among healthcare workers, including elevated BMI, fasting blood glucose, dyslipidemia, and hypertension. Behavioral risk factors such as physical inactivity, unhealthy dietary habits, tobacco use, and alcohol consumption are expected to be prevalent. Additionally, varying degrees of psychological distress, including depression, anxiety, and stress, are anticipated. Significant associations between cardiometabolic risk factors and demographic variables are expected to be identified.

Conclusion

The study findings provide valuable insights into the prevalence and determinants of cardiometabolic risk factors among healthcare workers in a rural setting. These insights can inform targeted interventions and public health strategies aimed at improving the cardiovascular health and overall well-being of healthcare workers, ultimately contributing to the enhancement of healthcare delivery and outcomes in rural areas.

## Introduction

Cardiometabolic diseases, encompassing conditions such as diabetes, hypertension, and dyslipidemia, pose a significant public health challenge globally, contributing to substantial morbidity and mortality rates [1]. In India, the burden of these diseases is particularly pronounced, with the prevalence steadily rising, fueled by urbanization, sedentary lifestyles, and shifting dietary patterns [2]. While considerable attention has been directed toward understanding and addressing cardiometabolic risk factors in the general population, there remains a dearth of research focusing on specific occupational groups, particularly healthcare workers, who are vulnerable to these conditions due to the nature of their profession [3].

Healthcare workers play a pivotal role in promoting health and managing diseases within their communities; however, the demanding nature of their work often leaves them susceptible to neglecting their health needs [4]. Factors such as long working hours, irregular mealtimes, and high levels of stress contribute to poor lifestyle habits among healthcare workers, placing them at an increased risk for cardiometabolic diseases [5]. Despite their critical role in public health, limited research elucidates the prevalence and determinants of cardiometabolic risk factors, specifically among healthcare workers in rural settings in India.

Therefore, this study protocol addresses this gap by comprehensively assessing cardiometabolic risk factors and their determinants among healthcare workers at Acharya Vinoba Bhave Rural Hospital (AVBRH), a tertiary care hospital located in rural Wardha, Maharashtra, India. By elucidating the prevalence and determinants of these risk factors, the study seeks to inform evidence-based interventions tailored to the unique needs of healthcare workers in rural settings, thereby contributing to improving their health and well-being.

## Materials and methods

Study design and methodology

The proposed study is a descriptive cross-sectional analysis that aims to evaluate cardiometabolic disease risk factors among healthcare workers at AVBRH, a tertiary care hospital in rural Wardha, Maharashtra. This design facilitates understanding the prevalence and determinants of these risk factors within this specific population.

Participants

The study includes a comprehensive demographic of healthcare workers, such as doctors, nurses, and class three and four employees, encompassing a range of roles within the hospital. All employees who voluntarily agree to participate and provide informed consent are included. To maintain the integrity of the study and ensure accurate physiological readings, pregnant women are excluded due to their unique metabolic profiles, which could skew results related to cardiometabolic risk factors.

Data collection procedure

Recruitment and Ethical Considerations

Before data collection commenced, the Institutional Ethics Committee of Datta Meghe Institute of Higher Education & Research reviewed and approved the study protocol to ensure compliance with ethical standards (approval number DMIHER(DU)/IEC/2024/138). Information sessions were organized for potential participants to inform them about the study’s objectives, the confidentiality of the data, and the voluntary nature of their participation. Following these sessions, informed consent was obtained from all participants. The data collection instruments are presented in Table 1, and the data collection steps are presented in Table 2.

**Table 1:** Data collection instruments DASS, Depression, Anxiety, and Stress Scale; ODK, Open Data Toolkit

Data collection instruments	Description
Modified WHO STEPS Instrument [6]	This tool is designed to collect standardized information on the major noncommunicable disease risk factors. It includes sections on tobacco use, diet, physical activity, and alcohol consumption to assess cardiometabolic risk factors.
Indian Diabetes Risk Score [7]	The Indian Diabetes Risk Score aids in identifying undiagnosed cases of diabetes based on several risk parameters. It provides a simple and effective method for screening individuals for diabetes risk.
DASS-21 Scale [8]	The DASS-21 is a questionnaire used to measure levels of depression, anxiety, and stress. It consists of 21 questions divided into three subscales to assess mental health status.
ODK Collect App [9]	ODK Collect is a mobile application designed to facilitate the electronic capture of data directly during interviews and examinations. It allows for efficient and accurate data collection in the field using digital forms.

**Table 2:** Data collection steps DASS, Depression, Anxiety, and Stress Scale

Data collection steps	Description
Sociodemographic and behavioral data collection	Participants completed a questionnaire detailing their age, gender, job role, marital status, dietary habits, physical activity, tobacco use, and alcohol consumption. This step is crucial for identifying behavioral risk factors associated with cardiometabolic diseases.
Anthropometric measurements	Measurements such as height, weight, and waist circumference are taken using standardized equipment. Blood pressure was measured three times using a calibrated sphygmomanometer, with the average of the last two readings recorded to ensure accuracy.
Biochemical measurements	Following a 10- to 12-hour fast, venous blood samples of hemoglobin A1C, thyroid profile, lipid profile, complete blood count, liver function tests, and renal function tests were collected. These samples were processed in the hospital’s central laboratory to ensure timely and accurate results.
Mental health assessment	The DASS-21 scale was administered to assess mental health status. This scale is divided into three subscales that help in evaluating the presence and severity of depression, anxiety, and stress.

Sample size

The sample size was calculated using a formula based on the proportion of the estimated population size. With an estimation error (d) of 5% and considering the total number of healthcare workers in the tertiary care hospital, the population size (N) is 1,650. The minimum sample size needed is 322.

Quality control and data management

To ensure the reliability and accuracy of the data, all measurement equipment was calibrated before the study and routinely checked throughout. Data was collected anonymously and managed securely with regular backups and restricted access. Additionally, pilot testing was conducted on 10% of the participants to refine the data collection process and address any potential issues before full-scale implementation.

Follow-up and reporting

Participants identified with risk factors or conditions such as hypertension or diabetes were referred for further medical evaluation and management. The preliminary results of the study were shared with both the participants and the hospital administration to ensure transparency and facilitate immediate health interventions. Ultimately, the findings were prepared for presentation at scientific forums and publication in peer-reviewed journals.

Outcome measures

The study is designed to evaluate a range of primary outcomes centered around the prevalence of key cardiometabolic risk factors among healthcare workers. These include anthropometric indicators such as BMI, waist circumference, and waist-to-hip ratio; biochemical parameters like fasting blood glucose, total cholesterol, low-density lipoprotein cholesterol, high-density lipoprotein cholesterol, and triglycerides; and blood pressure measurements to determine the prevalence of hypertension. Behavioral factors, including physical inactivity, dietary habits, tobacco use, and alcohol consumption, were also assessed. Additionally, the study investigated mental health status, particularly focusing on the prevalence and severity of depression, anxiety, and stress as measured by the Depression, Anxiety, and Stress Scale (DASS)-21 scale.

Statistical analysis

The initial statistical analysis stage involves a comprehensive review and cleaning of the data to ensure accuracy and completeness. This preparation includes handling missing data, eliminating outliers, and validating all data entries. Once cleaned, the dataset underwent a detailed statistical analysis. For descriptive statistics, the baseline characteristics of the study population were summarized. Categorical variables such as gender, job role, and smoking status were described using frequencies and percentages. Continuous variables like age, BMI, cholesterol, and fasting glucose levels were summarized using means and standard deviations. The normality of these continuous variables was assessed through the Kolmogorov-Smirnov test to determine the appropriate statistical tests to apply. In the inferential statistics phase, the Student’s t-test was employed to compare the means of normally distributed continuous variables across different groups (e.g., comparing males versus females or diabetic versus nondiabetic participants). The Mann-Whitney U test was used to distribute the data. The Chi-Square test examined relationships between categorical variables, such as the association between job role and the presence of hypertension. Logistic regression models were employed to assess the impact of multiple variables on binary outcomes, such as the presence or absence of hypertension. These models allowed us to identify significant predictors of cardiometabolic risk factors while controlling for potential confounders. Pearson or Spearman correlation coefficients were computed to explore relationships among continuous variables, such as the correlation between BMI and blood lipid levels. The results from these analyses were presented in detail using tables and figures to depict the distribution of risk factors and their associations with demographic and behavioral variables. The adjusted ORs and 95% CIs were reported from logistic regression analyses to highlight the strength and precision of the identified associations. All statistical analyses were conducted using R Studio software version 4.3.2, which provides a robust platform for handling and analyzing extensive datasets efficiently and transparently, and a p-value less than 0.05 was considered significant. This comprehensive approach to data analysis aims to provide a deep understanding of the distribution and determinants of cardiometabolic risks among healthcare workers, informing targeted interventions to improve their health and well-being.

## Results

The anticipated results of this study hold significant implications for understanding and addressing cardiometabolic risk factors among healthcare workers in rural settings. It is expected that a considerable proportion of participants will exhibit elevated levels of key risk indicators, including BMI, waist circumference, fasting blood glucose, and dyslipidemia. Moreover, behavioral risk factors such as physical inactivity, poor dietary habits, and tobacco or alcohol use are anticipated to be prevalent among the study population.

## Discussion

The expected prevalence of cardiometabolic risk factors among healthcare workers aligns with existing literature highlighting the elevated risk of noncommunicable diseases in this population [10]. The anticipated high prevalence of obesity, dyslipidemia, and hypertension underscores the urgent need for targeted interventions to promote healthy lifestyle behaviors and mitigate disease burden [11].

Behavioral risk factors such as physical inactivity and unhealthy dietary habits are expected to contribute significantly to the observed cardiometabolic risk profiles. These findings are consistent with global trends indicating a shift toward sedentary lifestyles and dietary patterns high in processed foods, which pose significant challenges to public health efforts [12]. The study’s anticipated findings on the mental health status of healthcare workers are particularly noteworthy. Previous research has identified high levels of psychological distress among healthcare professionals, attributed to factors such as heavy workloads, organizational stressors, and exposure to traumatic events [13]. Addressing mental health issues within the healthcare workforce is crucial not only for the well-being of individual workers but also for the delivery of quality patient care [14].

The expected associations between cardiometabolic risk factors and demographic variables underscore the importance of considering socio-demographic factors in public health interventions. Targeted strategies tailored to specific demographic groups may be more effective in addressing health disparities and promoting equitable access to healthcare services [15]. Methodological considerations, including the use of standardized assessment tools and rigorous statistical analysis, enhance the validity and reliability of the study findings. However, limitations such as self-reported data and potential selection bias should be acknowledged and addressed in future research endeavors.

## Conclusions

The anticipated findings of this study hold paramount significance for understanding and addressing the complex landscape of cardiometabolic risk factors among healthcare workers in rural areas. With expected high prevalence rates of obesity, dyslipidemia, hypertension, and behavioral risk factors such as physical inactivity and unhealthy dietary habits, the study sheds light on the urgent need for targeted interventions to mitigate these risks and promote better cardiovascular health among this vital workforce. Moreover, the anticipated associations between cardiometabolic risk factors and demographic variables emphasize the importance of tailored approaches that consider age, gender, job role, and socioeconomic status in health promotion strategies. By recognizing and addressing the multifaceted challenges faced by healthcare workers, including psychological distress, this study contributes valuable insights to inform evidence-based interventions aimed at improving the overall well-being and quality of life of healthcare professionals in rural settings. Ultimately, the findings underscore the critical role of proactive public health initiatives and workplace interventions in safeguarding the health of those dedicated to caring for others.
